# A Worm-like Crawling Soft Robot with Pneumatic Actuators Based on Selective Laser Sintering of TPU Powder

**DOI:** 10.3390/biomimetics7040205

**Published:** 2022-11-20

**Authors:** Tianhao Du, Lechen Sun, Jingjing Wan

**Affiliations:** College of Design and Engineering, National University of Singapore, Singapore 117575, Singapore

**Keywords:** soft robotics, bioinspired crawling robot, pneumatic actuators, finite element analysis (FEA), selective laser sintering (SLS) technology

## Abstract

Soft robotics is one of the most popular areas in the field of robotics due to advancements in bionic technology, novel materials, and additive manufacturing. Existing soft crawling robots with specific structures have a single locomotion mode and cannot complete turning. Moreover, some silicone-based robots lack stiffness, leading to unstable movements especially when climbing walls, and have limited environmental adaptability. Therefore, in this study, a novel crawling soft robot with a multi-movement mode and high environmental adaptability is proposed. As the main structure of the robot, pneumatic single-channeled and double-channeled actuators are designed, inspired by the worm’s somite expansion and contraction. Model-based methods are employed to evaluate and analyze the characteristics of the actuators. By the application of selective laser sintering technology and thermoplastic polyurethane (TPU) material, the fabricated actuators with an auxetic cavity structure are able to maintain a certain stiffness. Via the coordination between the actuators and the suckers, two locomotion modes—straight-line and turning—are realized. In the testing, the speed of straight-line crawling was 7.15 mm/s, and the single maximum turning angle was 28.8 degrees. The testing verified that the robot could realize crawling on flat ground, slopes, and smooth vertical walls with a certain stability and equipment-carrying capacity. This research could lay the foundation for subsequent applications, including large tank interior inspections, civil aviation fuselage and wing inspections, and wall-cleaning in high-rise buildings.

## 1. Introduction

Soft crawling robots [1,2] are widely used in medical [3] and industrial [4] applications and daily life due to their superb environmental adaptability and geometric access capabilities. The design of most soft crawling robots is inspired by various types of crawling organisms that have evolved in nature over billions of years and have completely adapted to their environments. Studying how animals move around their complex, unpredictable environments could provide valuable insights into emerging robotics applications in medicine, search and rescue, disaster response, and humans [5]. For example, organisms such as inchworms and worms can be well adapted to crawling on columnar objects such as tree trunks. Koh et al. [6] mimicked the motion of inchworms with SMA and designed the Omegabot to achieve crawling on tree trunks. Khan et al. [7] designed the iCrawl robot by utilizing inchworm morphology and evolutionary behavior to adapt to and climb complex environments such as the outer surfaces of metal tubes. Creatures such as fish are well-adapted to the underwater environment and achieve movement. Romano et al., inspired by the Karan swimmers, used the magnetic field interaction of permanent magnets to convert the motor’s rotating motion into an oscillating motion to achieve its motion in water [8]. Inspired by carangiform fish, Liu et al. designed a multi-articular robotic fish that realized two basic motion modes: straight cruise and C-shaped sharp turn [9].

Based on the motion patterns of different crawling organisms, the current, relatively mature soft robot motion mechanisms include inchworm-like motion, worm-like motion, and snake-like meandering motion.

The body of an inchworm can be divided into three parts: trunk, front end, and rear end. When the inchworm moves, it first fixes the front end, bends the trunk to form an Ω shape, then fixes the rear end, and then relaxes the trunk to move forward. Since the posture formed by the body in inchworm-like motion is similar to the symbol Ω, the inchworm-like motion movement is also called Omega motion [10]. Based on the inchworm motion, Zhang et al. [11] designed a soft crawling robot to realize the transition from a flat surface to a slope. The same design idea is also used for the pneumatic soft robot PISROB designed by Xie et al. [12], which can crawl on smooth flat plates as well as round rods.

The carapace of worms, earthworms, and other annelids is a typical static skeletal structure. In the case of the earthworm, for example, its muscles are oblique and consist of the annulus and longitudinal muscles. When the longitudinal muscle of a somite contracts, the diameter of the somite increases and the length decreases; when the circular muscle contracts, the diameter of the somite decreases and the length increases. Earthworms achieve forward motion through traveling waves generated by muscle contractions between individual body segments [13]. This type of movement is called worm-like motion. Qin et al. [14] designed a worm-like soft robot based on this property, and it can walk in a straight line, turn, and even crawl on vertical walls. Zhang et al. [15] also designed a soft robot that can adapt to complex pipeline environments based on a worm-like motion pattern.

Snakes have a slightly more complex locomotion mechanism than the above two animals. The basic movement of snakes is called meandering, and all snakes can crawl forward in this manner. When crawling, the snake’s body forms a horizontal wave-like bend on the ground so that the back side of the bend applies force to the rough ground, and the reaction force from the ground pushes the snake’s body forward [16]. Based on the meandering motion of snakes, Cao et al. [17] proposed a new mechanism for the slithering motion of snake-like soft robots. They verified the slithering motion mechanism of snakes using finite element method (FEM) simulations. Branyan, C et al. [18] presented an entirely soft snake robot designed to implement the prerequisite shape space for slithering gaits.

For crawling soft robots, the anchoring method also affects the robot’s ability to adapt to complex environments and perform complete corresponding movements. Currently, commonly used anchoring devices include friction feet, grippers, vacuum suction cups, etc. The friction foot relies on a specific structure that increases the friction between the contact surface to achieve anchoring. Liu et al. [19] studied a new design integrating a single pneumatic actuator and a negative Poisson’s ratio structure to change the friction force by elongating the pneumatic actuator and changing the negative Poisson’s ratio structure at both ends to achieve the elongation motion and anchoring motion of the soft robot in creeping motion. The design of crawling feet can reduce the number of actuators. Nevertheless, it is challenging to achieve crawling on slopes or walls to overcome gravity due to the poor stability of the anchoring method with applied friction.

Grippers include soft grippers and rigid grippers. The soft caterpillar soft robot studied by Shane et al. [20] applied a passive grasping, active-release soft gripper to achieve climbing on tree branches. Lam et al. [21] studied the tree bot, which used the active clamping of a rigid-body gripper to achieve crawling on a tree trunk. Collectively, the soft gripper can better adapt to different shapes of rods, and the rigid gripper can provide more clamping force. Nonetheless, the anchoring method of applying the gripper is only suitable for climbing columnar objects such as pipes and tree trunks and does not apply to vertical surfaces.

The sucker adopts the principle of a vacuum and can use the vacuum’s negative- pressure “adsorption” surface to achieve the purpose of anchoring. Vacuum adsorption has the advantages of being clean, allowing smooth adsorption, and being reliable, and it does not damage the surface of the anchored objects, commonly used in the anchoring of smooth walls. Huang et al. [22] realized the anchoring of a multimodal soft robot using vacuum suction cups to climb vertical walls and transition from one surface to another.

The actuator is an essential part of the soft robot. The current drive methods of soft robots mainly include pneumatic [23], dielectric elastomer (DE) [24], wire drive [25], shape memory alloys (SMA) [26], etc. Due to the simple control, rapid response, and low manufacturing cost of pneumatic actuators, they are still the mainstream driving method in soft crawling robots. Pneumatic actuators are generally manufactured using hyperelastic materials such as silicone rubber by injection molding, fused deposition modeling (FDM), stereolithography appearance (SLA), and direct ink writing (DIW) [27]. However, due to the low stiffness of the flexible actuators manufactured in these ways, they are subject to gravity. They are not suitable for working on large, inclined slopes or on vertical walls. 

After a comprehensive analysis of the locomotion mode, anchoring mode, actuator structure, manufacturing, and material issues, this paper presents a worm-like pneumatic soft crawling robot. With the coordination of the actuators and suckers, two modes of locomotion can be realized: straight-line and turning, thereby resolving one of the drawback of the existing soft robots, which is that they only possess a single motion mode. The actuators are innovatively manufactured by selective laser sintering of TPU powder with a Shore hardness of 90A, which improves the manufacturing accuracy and dramatically increases the stiffness of the actuator, reducing the impact of gravity on the actuator during movement while ensuring the hyperelasticity of the actuators. Hence, this robot can adapt to complex environments, such as crawling on slopes and vertical walls, and not just on flat ground. Furthermore, compared with the existing soft crawling robots, this robot has a faster moving speed and higher equipment-carrying capacity. It has high application value in the inspection of large tank interiors, civil aviation fuselages and wings, and in glass curtainwall-cleaning in high-rise buildings.

## 2. Robot Design

### 2.1. Overall Structural Design of the Soft Crawling Robot

In nature, the movement of worms requires the coordination of longitudinal and transverse muscles. To simplify this process, hollow and deformable actuators can be designed. By inflating the actuators, deformation occurs, which is then converted into the movement of the robot. This design can successfully mimic the properties of the worm’s somite expansion and contraction. Therefore, a soft crawling robot with these pneumatic actuators as its main feature is proposed in this study.

**Figure 1:**
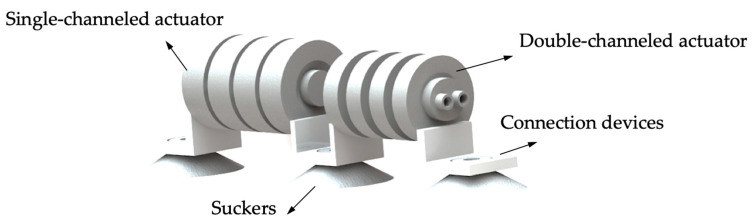
The overall design of the soft crawling robot.

As shown in Figure 1, the soft crawling robot consists of four main parts: the single-channeled actuator, double-channeled actuator, connecting devices, and suckers. Although there are some limitations in using suckers as the anchoring method for this robot, such as a limited application environment, they are inexpensive, simple to control, and have strong anchoring ability, which enables the robot to crawl steadily on flat ground and walls. The connecting device is bonded to the actuator, and the suckers are fixed by interference fit.

### 2.2. Structure of Single-Channeled Actuator 

The purpose of the single-channeled actuator is to allow the soft crawling robot to complete an elongation movement, which can be translated into a straight movement by anchoring the robot’s feet. The constituent materials of the actuator have to deform considerably under air pressure, and the structure of the actuator needs to ensure anisotropy of the deformation, i.e., deformation in one direction.

**Figure 2:**
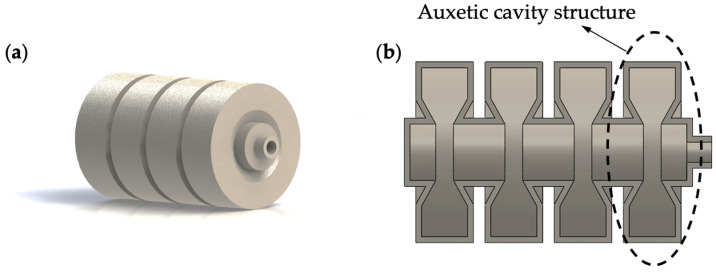
Single-channeled actuator. (**a**) Three-dimensional view; (**b**) sectional view.

Three-dimensional and cross-sectional views of the single-channeled actuator are shown in Figure 2. The single-channeled actuator adopts a bellows-like structure, similar to the actuator structure developed by Z. Jiao et al. [28], and large stiffness characterizes the bellows structure at the ridges and valleys of the bellows, with small stiffness in the direction of the undulation of the bellows. Therefore, the bellows structure can achieve large deformation in the axial direction and small deformation in the radial direction under air pressure. When the air pressure decreases, the single-channeled actuator will return to its initial state due to the material’s elasticity. In addition, the bellows structure has the characteristics of fast braking and low working air pressure, which helps to improve the robot’s crawling speed [29]. It is worth mentioning that durability is a critical concern for pneumatic actuators that rely on material deformation. In Figure 2b, it can be seen that the single-channeled actuator is not a regular bellows structure; at the bottom of each cavity, there is a concave inward structure, which we can call the auxetic cavity structure. In this case, when inflated, the effect of the axial deformation of the actuator is better than that of the standard cavity structure. Additionally, after inflation, the stress on the edge part of the cavity structure will be decreased, reducing the possibility of local fatigue and plastic deformation, which improves the actuator’s service life.

### 2.3. Structure of Double-Channeled Actuator Design

**Figure 3:**
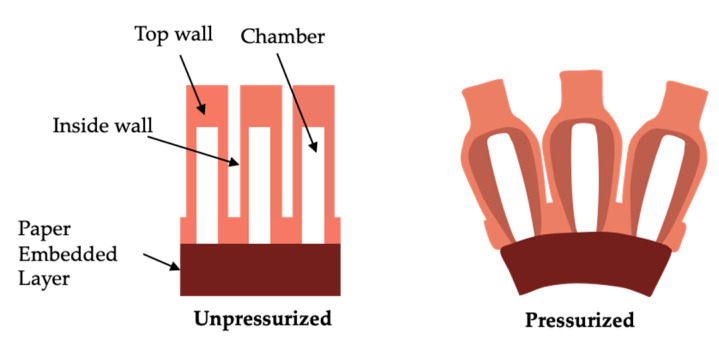
Pneumatic Network [30] by Harvard University.

**Figure 4:**
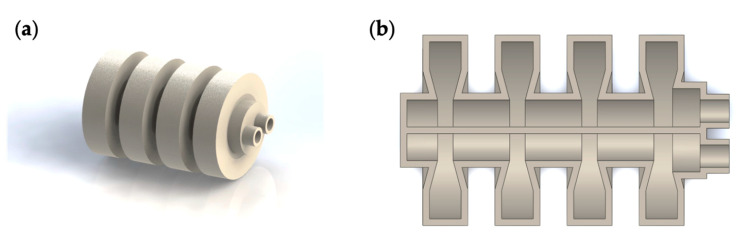
Double-channeled actuator. (**a**) Three-dimensional view; (**b**) sectional view.

The design of the double-channeled actuator is inspired by the Pneumatic Network (PN actuator) developed at Harvard University [30], as shown in Figure 3. When gas is passed into the PN actuator, the actuator deforms considerably, causing the cavities to squeeze each other and causing the actuator to bend. Suppose that two similar PN actuators are allowed to be combined by sharing a bottom surface, as shown in Figure 4. In this case, this double-channeled actuator can bend in two directions by inflating to different channels and enabling the soft crawling robot to complete turning motions. The PN actuator has a restriction layer on the bottom side. The limiting layer was ignored to form the double-channeled actuator in one piece. 

Similarly, the auxetic cavity structure was also designed in the double-channeled actuator. In addition, when the double-channeled actuator is bending, if the bending angle is relatively large, the two adjacent cavities will come into contact and generate forces. Therefore, this should be avoided when choosing the appropriate structural parameters and the inflation pressure.

### 2.4. Locomotion Gait Design of the Robot

**Figure 5:**
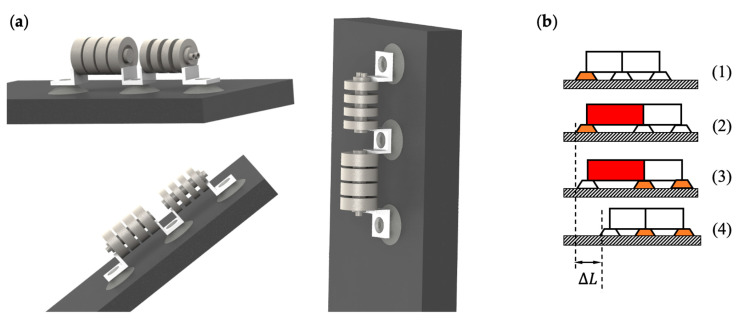
Straight-line locomotion. (**a**) Performed on flat ground, slopes, and vertical walls. (**b**) Gait design.

Two types of locomotion gaits were designed for the soft crawling robot based on its structural characteristics, including straight walking and turning. Theoretically, straight-line locomotion can be performed on flat ground, slopes, and even vertical walls, respectively, as shown in Figure 5a. The straight-line locomotion is accomplished by deformation of the single-channeled actuators, together with the stable anchoring and release of the suckers, to ensure that the robot movement does not slip back.

Figure 5b shows the gait design for the straight-line locomotion of the soft crawling robot. For simplicity, the double-chamber actuator is ignored in the figure. Red indicates that the actuator is in operation, and orange indicates that the suckers are in service. The soft crawling robot’s straight-line locomotion gait design is similar in the three environments, with four steps. First, the rear-end sucker works and adsorbs on the ground (Figure 5b. (1)); then, an air pump pumps air into the single-channeled actuator to deform and elongate it, generating a forward driving force. As the rear-end sucker adsorbs on the ground, the front-end sucker slides forward under the driving force to overcome the friction (Figure 5b. (2)); after this, the front-end sucker also anchors on the ground (Figure 5b. (3)); finally, the rear-end sucker stops working. At the same time, the air inside the actuator is released. The actuator returns to its original state under the elastic recovery force, thus producing a moving distance of one step for the robot (Figure 5b. (4)). According to the above cyclic motion, the soft robot continuously expands and elongates and returns to its original state, thus achieving straight-line locomotion.

**Figure 6:**
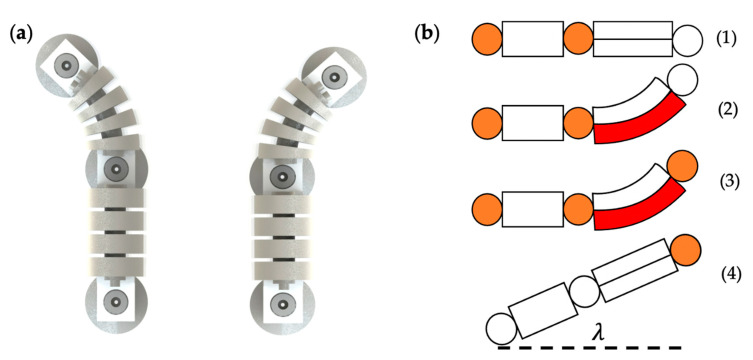
Turning locomotion. (**a**) Left turn and right turn. (**b**) Gait design.

The turning locomotion of the soft crawling robot is mainly realized by the bi-directional bending of the double-channeled actuator, as shown in Figure 6a. When turning, the air pump passes air into one channel of the actuator, and the actuator bends and deforms due to the different air pressure on both sides, causing the soft robot to turn to one side.

Figure 6b demonstrates the gait design of the turning locomotion of the soft crawling robot. The middle suckers work in the same state as the rear-end sucker during the turning process. In the left-turning gait, first, the rear-end sucker anchors on the ground (Figure 6b. (1)); the air pump fills a certain amount of gas into the right channel, which causes the double-channeled actuator to bend to the left (Figure 6b. (2)); after the actuator bends at a certain angle, the front-end sucker also starts to work and anchors on the ground (Figure 6b. (3)); finally, the rear-end sucker stops working, the gas is released from the actuator, and the actuator returns to its original state, driving the whole robot to turn λ degrees to the left (Figure 6b. (4)). In the right-turning gait, the difference is that gas is filling into the left channel.

## 3. Modeling of Soft Pneumatic Actuators

### 3.1. Nonlinear Hyperelastic Model of the Material

The soft actuators designed in this paper are composed of thermoplastic polyurethane (TPU). TPU is a hyperelastic material [31] that exhibits elastic deformation under an external load and can recover its original shape after it disappears. It has the characteristics of geometric nonlinearity and material nonlinearity. With the in-depth research on hyperelastic materials, hyperelastic constitutive models represented by the Ogden, Yeoh, and Mooney–Rivlin models have been developed [32].

**Figure 7:**
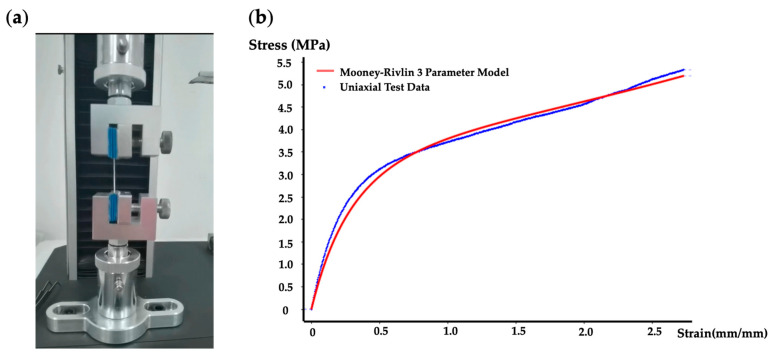
Characterization of hyperelastic properties of TPU materials. (**a**) Uniaxial tensile test. (**b**) Comparison between uniaxial test data and the fitted Mooney–Rivlin three-parameter model.

To find the model parameters and the most suitable hyperelastic model for the TPU material we used, it was first required to perform uniaxial tensile tests with ISO 37 as the standard, as shown in Figure 7a. Test specimens were fabricated by selective laser sintering technology, the same technology used to manufacture the actuators.

The sum of square error method was used to determine the best hyperelastic model. The Mooney–Rivlin model was demonstrated to be the best method to characterize the hyperelastic properties of this TPU material. Moreover, for the stress–strain curve with one inflection point, the three-parameter model was suitable [5]. Figure 7 shows the stress–strain values obtained from uniaxial tensile tests of the material and the curves fitted with the Mooney–Rivlin three-parameter model. The strain energy density function [5] W for the Mooney–Rivlin three-parameter model is

(1) $$W=C_{10}\left(\bar{I_{1}}-3\right)+C_{01}\left(\bar{I_{2}}-3\right)+C_{11}\left(\bar{I_{1}}-3\right)\left(\bar{I_{2}}-3\right)+\frac{1}{D_{1}}{\left(J-1\right)}^{2}$$

where $\bar{I_{2}}$ are the first and second invariants of the strain deviator, and J is the elastic volume ratio; when the material is considered incompressible, J=1, $C_{10}$, $C_{01}$, $C_{11}$, and $D_{1}$ are material parameters. The parameter values of this TPU material are $C_{10}=0.074$ mPa, $C_{01}=2.066$ mPa, $C_{11}=0.196$ mPa, $D_{1}=0$.

### 3.2. Mathematical Model for Actuator Structures

#### 3.2.1. Single-Channeled Actuator

**Figure 8:**
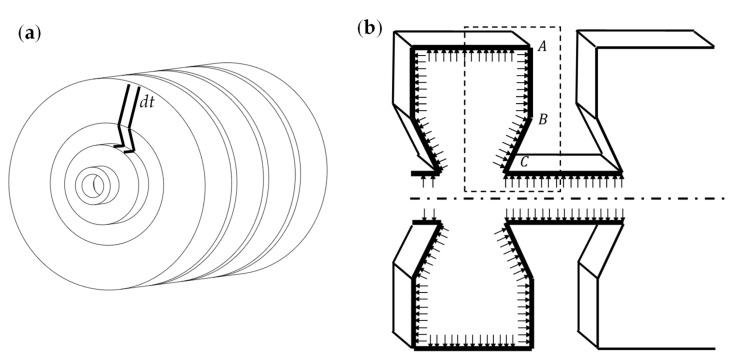
Actuator slice analysis. (**a**) Slice position. (**b**) Key structure A−B−C.

As shown in Figure 8a, using the microelement method, the actuator is divided into an infinite number of slices of thickness dt. The structure A−B−C in Figure 8b is the critical structure that determines the elongation of the actuator, and the internal force of each slice is the same for the air pressure and is evenly distributed inside the actuator’s cavity. We can obtain the deformation of the entire actuator by discussing the deformation of the structure A−B−C separately, and, in the subsequent analysis, we refer to the structure A−B−C as a ‘joint’ of the actuator. The slice of the actuator needs eight joints with the same shape to work together to complete an elongation movement, and the force and deformation of each ‘joint’ are precisely the same.

We made the following assumptions before building the mathematical model.

1. In the preliminary experiment, the radial expansion of the actuator is much smaller than the actuator’s radius, so the actuator’s radial expansion is ignored—that is, the actuator does not have any deformation in the radial direction (H is the outer diameter of the actuator and remains the same).

2. The lengths of line segment AB and line segment CD do not change in any way and are never bent without considering the extreme deformation, which is

(2) $$AB=AB^{\prime }$$

(3) $$BC=BC^{\prime }$$

3. We fix point A to facilitate the calculation of the displacement of the actuator.

**Figure 9:**
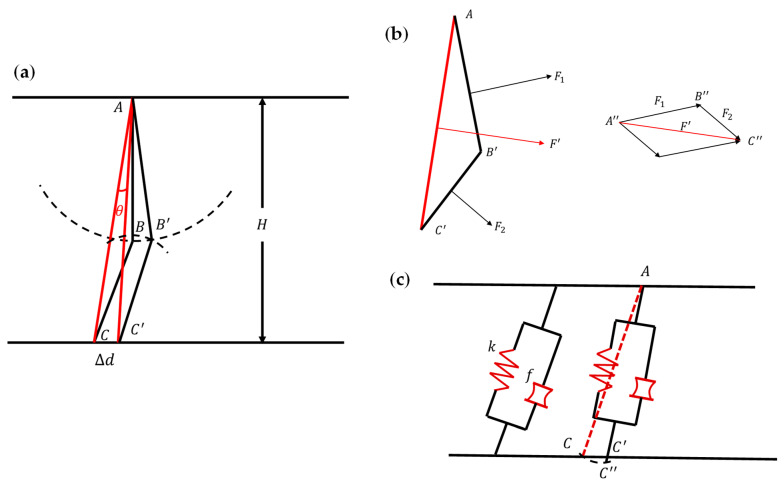
Actuator ‘joint’ mathematical model. (**a**) Deformation of a ‘joint’ in one movement. (**b**) Air pressure work equivalent model. (**c**) Mechanical equivalent model of the structure A−B−C.

As shown in Figure 9a, we first discuss the deformation of a ‘joint’. It is stipulated that when the air pressure is p, the angle between the starting position AC and the stable position $AC^{\prime }$ is θ. The work performed by the air pressure inside the actuator $W_{a}$ is equal to the energy stored Q in the deformation of the structure A−B−C:

(4) $$W_{a}=E$$

In order to calculate the air pressure work, we equalize the forces of AB and BC to the force of AC in Figure 9b.

(5) $$\left|\vec{F_{1}}\right|=p\left|AB_{1}\right|dt$$

(6) $$\left|\vec{F_{2}}\right|=p\left|B_{1}C_{1}\right|dt$$

(7) $$\vec{F_{1}}+\vec{F_{2}}=\vec{F^{\prime }}$$

Thus, according to the geometric relationship, $\Delta AB^{\prime }C^{\prime }\sim \Delta A^{\prime \prime }B^{\prime \prime }C^{\prime \prime }$

(8) $$\left|\vec{F^{\prime }}\right|=p\left|AC_{1}\right|dt$$

(9) $$\left|AC_{1}\right|=\frac{H}{\cos\left(∠CAB-\theta \right)}$$

The process of air pressure working on AB and BC can be equivalent to the work performed by force $\vec{F^{\prime }}$ on AC:

(10) $$dW_{a}=M\times d\theta =\left|\vec{F^{\prime }}\right|\times \frac{1}{2}\left|AC_{1}\right|\times d\theta =\frac{1}{2}p{\left[\frac{H}{\cos\left(∠CAB-\theta \right)}\right]}^{2}dtd\theta$$

(11) $$W_{a}=\int_{0}^{\alpha }dW_{a}=\frac{pH^{2}dt}{2}\int_{0}^{\alpha }{\left[\frac{1}{\cos\left(∠CAB-\theta \right)}\right]}^{2}d\theta$$

(12) $$W_{a}=\frac{pH^{2}dt}{2}\tan\left(∠CAB\right)-\frac{pH^{2}dt}{2}\tan\left(∠CAB-\alpha \right)$$

As shown in Figure 9c, $\left|AC\right|$ is smaller than $\left|AC^{\prime }\right|$, and compression amount $\Delta l=\left|C^{\prime }C^{\prime \prime }\right|$. We use the compression amount to calculate the energy from the system deformation. AC is equivalent to a 2-element model including a thrust element and a spring element in parallel whose parameters are f,k. The values of f and k could be obtained from the test.

(13) $$E=E_{s}+E_{t}=\frac{k{\left(\Delta l\right)}^{2}dt}{2}+f\Delta ldt$$

According to the geometric relationship,

(14) $$\Delta l=\left|AC^{\prime }\right|-\left|AC\right|=\frac{H}{\cos\left(∠CAB\right)}-\frac{H}{\cos\left(∠CAB-\alpha \right)}$$

(15) $$\Delta d=Htan\left(∠CAB\right)-Htan\left(∠CAB-\alpha \right)$$

Combined with the above formulas,

(16) $$\begin{array}{l} \frac{k{\left[\frac{H}{\cos\left(∠CAB\right)}-\frac{H}{\cos\left(∠CAB-\alpha \right)}\right]}^{2}}{2}+f\left[\frac{H}{\cos\left(∠CAB\right)}-\frac{H}{\cos\left(∠CAB-\alpha \right)}\right]\\ =\frac{pH^{2}}{2}\tan\left(∠CAB\right)-\frac{pH^{2}}{2}\tan\left(∠CAB-\alpha \right) \end{array}$$

(17) $$p=H\left(\alpha \right)=\frac{k{\left[\frac{H}{\cos\left(∠CAB\right)}-\frac{H}{\cos\left(∠CAB-\alpha \right)}\right]}^{2}+2f\left[\frac{H}{\cos\left(∠CAB\right)}-\frac{H}{\cos\left(∠CAB-\alpha \right)}\right]}{H^{2}\left[\tan\left(∠CAB\right)-\tan\left(∠CAB-\alpha \right)\right]}$$

Finally, ΔD is the total elongation, and we then obtain the relationship between ΔD and p:

(18) $$\Delta d=Htan\left(∠CAB\right)-Htan\left[∠CAB-H^{-1}p\right]$$

(19) $$\Delta D=8\Delta d=8Htan\left(∠CAB\right)-8Htan\left[∠CAB-H^{-1}p\right]$$

#### 3.2.2. Double-Channeled Actuator

**Figure 10:**
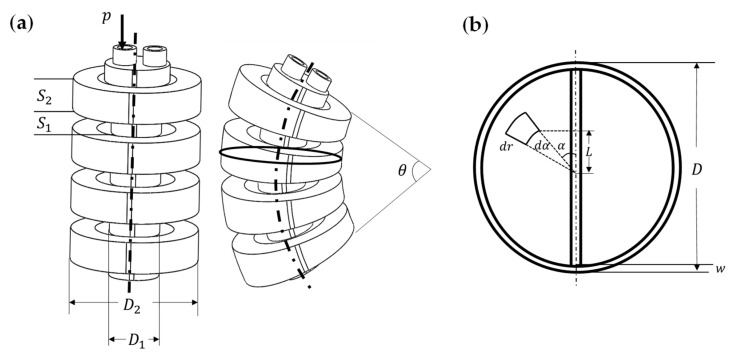
Mathematical model of the bending actuator. (**a**) Bending angle θ when left cavity’s air pressure is p. (**b**) The cross-section of the actuator.

As shown in Figure 10a, when inflating the left cavity of the bending actuator, it bends in the right direction. Moreover, to analyze the relationship between the bending angle θ and the air pressure of the cavity, we regard the bending actuator as a cantilever beam. The actuator is divided into seven beams, of which three beams have section radius $D_{1}$ and four beams have section radius $D_{2}$. The bending angles of these seven beams are calculated and superimposed to obtain the bending angles of the actuator. According to the Euler–Bernoulli principle,

(20) $$\theta_{1}=\frac{M_{1}S_{1}}{EI_{1}}$$

(21) $$\theta_{2}=\frac{M_{2}S_{2}}{EI_{2}}$$

(22) $$\theta =4\theta_{2}+3\theta_{1}$$

where $M_{1}$ and $M_{2}$ are the bending moment, and $S_{1}$ and $S_{2}$ denote the lengths of two different beams, respectively. E is the Young’s modulus.

Using the micro-element method, a very short beam is intercepted, and the moment of inertia of the beam section I can be regarded as the moment of inertia of a ring. Figure 10b shows the cross-section of the actuator. $I_{1}$ and $I_{2}$ are the cross-section moments of inertia of different diameters $D_{1}$ and $D_{2}$, and then

(23) $$I_{1}=\frac{\pi \left[D_{1}{}^{4}-{\left(D_{1}-2w\right)}^{4}\right]}{64}$$

(24) $$I_{2}=\frac{\pi \left[D_{2}{}^{4}-{\left(D_{2}-2w\right)}^{4}\right]}{64}$$

where w denotes the thickness of the actuator.

On the cross-sections of the left cavity, a force area micro-element is taken, and we can obtain

(25) dA=rdrdα

(26) dF=dAp=prdrdα

(27) $$dM=dFL=pr^{2}\sin\alpha drd\alpha$$

(28) $$\;M=\int_{0}^{\pi }\int_{0}^{\frac{D}{2}-w}pr^{2}\sin\alpha drd\alpha =\frac{4}{3}p{\left(\frac{D}{2}-w\right)}^{3}$$

Combined with the above formula, we can obtain the bending angle of the actuator:

(29) $$\theta =\frac{16p{\left(\frac{D_{2}}{2}-w\right)}^{3}S_{2}}{3EI}+\frac{4p{\left(\frac{D_{1}}{2}-w\right)}^{3}S_{1}}{EI}$$

From the mathematical models of the single- and double-channeled actuators, we find that geometric factors such as the diameter and thickness of the actuators affect their deformation characteristics. In the process of modeling the mathematical model, the traditional theory of rigid body mechanics is not applicable because the deformation of the actuator material is not negligible. Therefore, we introduced a rheological-mechanical equivalent model to solve this problem. However, some parameters in this model, such as f, k, are the material’s own properties. To obtain the specific values of these parameters, complex tests are required. As a result, the mathematical model is only suitable for qualitative analysis. In order to describe the deformation of the actuators visually and accurately, it is necessary to introduce a finite element model that can combine both the structural properties and material properties.

### 3.3. Friction Model of the Robot

**Figure 11:**
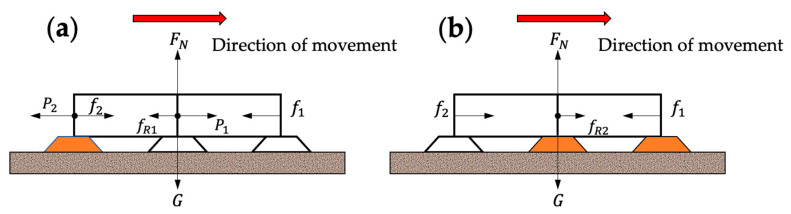
Force analysis of straight-line locomotion. (**a**) When the front-end and middle suckers move. (**b**) When the rear end sucker moves.

The friction model is the key in the analysis of forces during robot motion: the robot needs to overcome the friction generated by contact with the ground in order to move; the robot has to be anchored to the ground by the friction caused by the suction of the suction cups, thus ensuring the stability of the motion. Because of the soft crawling robot’s irregular shape and complex internal structure, this paper analyzes its critical state qualitatively. Figure 11 shows the force situation when the front-end sucker of the robot is moving and the rear-end sucker is moving, and the orange color means the suckers are working, which corresponds to the gait states of (1) and (2) in Figure 5b, respectively. In Figure 11a, the robot is subjected to the friction force $f_{1}$ between the front sucker (including the middle sucker) and the ground, the friction force $f_{2}$ between the rear-end sucker and the ground, the pressure $P_{1},\;P_{2}$ of the gas on the axial direction of the single-channeled actuator (ignoring the pressure in the radial direction), the resistance $f_{R1}$ that prevents the deformation of the actuator, the gravity G, and the support force $F_{N}$ of the ground on the robot. In order for the rear-end sucker to be fixed and the actuator to move the front-end sucker, the following conditions need to be met:

(30) $$\left\{\begin{array}{l} f_{1}+f_{R1}<P_{1}S_{act}\\ f_{2}>P_{2}S_{act} \end{array}\right.$$

Additionally, variables such as friction and support forces have the following relationships:

(31) $$\left\{\begin{array}{l} G=F_{N}\\ f_{1}=\frac{2\mu F_{N}}{3}\\ f_{2}=\frac{\mu F_{N}}{3}+\mu P_{atm}S_{suc\ker} \end{array}\right.$$

where $S_{act},\;S_{suc\ker}$ are the sum of the cross-sectional area of the cavities and the ground area of the sucker, respectively. μ is the coefficient of friction between the sucker and the ground, and $P_{atm}$ is the atmospheric pressure.

In Figure 11b, the actuator is no longer subject to air pressure because the gas in the actuator is released. With the front-end sucker fixed, the driver contracts forward by its own elasticity, thus overcoming the friction of the ground and then pulling the rear-end sucker forward. This process needs to satisfy the following equation:

(32) $$\left\{\begin{array}{l} f_{1}>f_{R2}\\ f_{2}<f_{R2} \end{array}\right.$$

In this case,

(33) $$\left\{\begin{array}{l} G=F_{N}\\ f_{1}=\frac{2\mu F_{N}}{3}+\mu P_{atm}S_{suc\ker}\\ f_{2}=\frac{\mu F_{N}}{3} \end{array}\right.$$

where $f_{R2}$ the restoring force of the actuator, which is related to the amount of deformation of the material.

In addition, if the robot moves on a slope or vertical wall, the suckers need to be considered for their adsorption to prevent the robot from slipping. In this paper, only the most dangerous case is analyzed, i.e., the gait shown in Figure 5b. (2) when the robot is crawling in a straight line on the wall. This is because, at this point, the lowest sucker carries the weight of the entire robot and is subject to the downward thrust of the actuator. If the robot can operate normally in this case, then the robot can move normally when performing other gaits or when it is on a slope.

**Figure 12:**
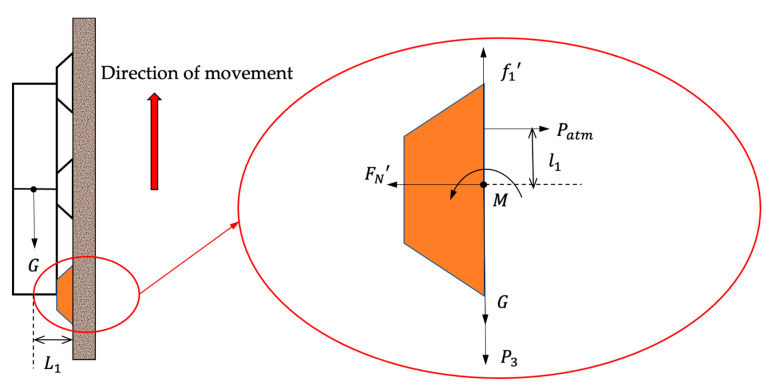
Force analysis of the lower-end sucker during the gait (2) of the robot when wall crawling.

The lower sucker, in the state shown in Figure 12, is subjected to friction, robot gravity G and thrust $P_{3}$ from the actuator due to air pressure, bending moment M from gravity and thrust, fraction force ${f_{1}}^{\prime }$ atmospheric pressure $P_{atm}$ and support forces $F_{N}{}^{\prime }$. In order for the robot to remain stable on the wall, the following conditions need to be met:

(34) $$\left\{\begin{array}{l} M<(P_{atm}-F_{N}{}^{\prime })l_{1}\\ f_{1}{}^{\prime }>G+P_{3}S_{act} \end{array}\right.$$

Additionally,

(35) $$\left\{\begin{array}{l} M=(P_{3}S_{act}+G)L_{1}\\ f_{1}{}^{\prime }=\mu F_{N}{}^{\prime } \end{array}\right.$$

Although accurate values cannot be obtained due to the complexity of the robot structure and materials, by establishing the force analysis based on the friction model, it can be found that the air pressure of the actuator and the suction force of the suckers are the keys to the efficient and stable movement of the whole robot system. When the suckers are not working, the friction between the suckers and the floor is small compared to the driving force of the actuators, so the robot is able to move. When the suckers are working, the friction between the suction cups and the floor or wall can be large, avoiding unstable robot locomotion.

### 3.4. Finite Element Method Model

**Figure 13:**
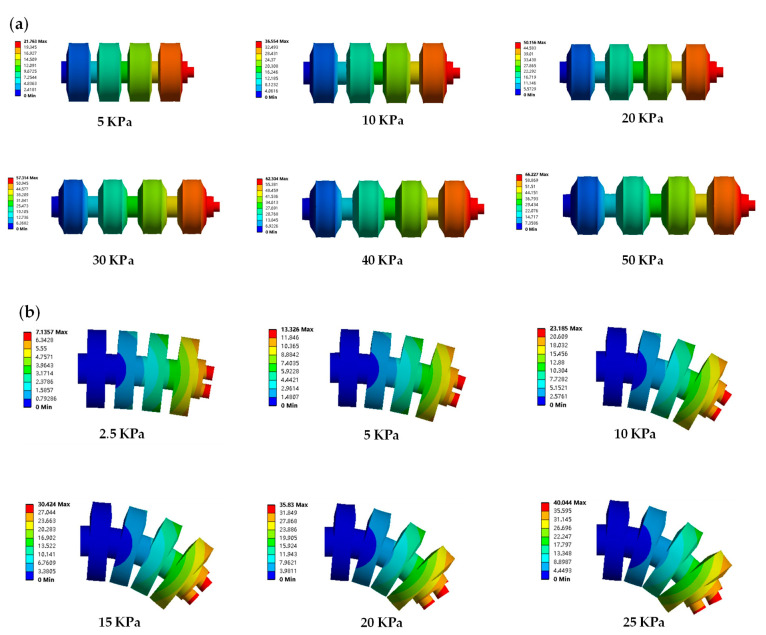
Deformation results in ANSYS Workbench. (**a**) Single-channeled actuator under the pressure of 5-30 KPa. (**b**) Double-channeled actuator under the pressure of 2.5–25 KPa.

The finite element analysis (FEA) method can integrate materials and structures to characterize soft single- and double-channeled actuators. The finite element simulation using ANSYS Workbench resulted in different states of the actuators at different air pressures. Workbench has various hyperelastic models embedded, and the TPU material used can be defined using the parameters of the Mooney–Rivlin three-parameter model. For the structures, a 2 mm tetrahedral mesh is divided. After different air pressures are applied inside the actuators, the deformation results for the single- and double-channeled actuators are obtained by deriving the finite element simulation model, as shown in Figure 13.

It is to be noted that if the air pressure in one channel of the double-channeled actuator continues to increase, the two adjacent cavities will collide. Thus, for the double-channeled actuator, the air pressure should be kept within 20 KPa. In addition, large stresses inside the actuator can cause it to fracture or have a reduced service life. Taking a single-channeled actuator as an example, the simulation results showed that the von Mises stresses of the actuator were 0.836 MPa and 1.161 MPa at air pressures of 30 KPa and 50 KPa, respectively.

**Figure 14:**
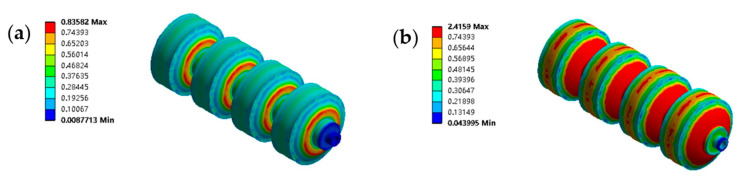
Design revisited for the auxetic cavity structure. (**a**) The von Mises stress of the single-channeled actuator with the auxetic cavity structure under 30 KPa air pressure. (**b**) The von Mises stress of the double-channeled actuator with the auxetic cavity structure under 100 KPa air pressure.

The finite element simulation results can illustrate why the auxetic cavity structure was designed. In the tests, we selected 50–60 mm as the step length of the robot locomotion (elongation of the single-cavity actuator). For the actuator with the auxetic cavity structure, 30 KPa air pressure is available, while, for the actuator without the auxetic cavity structure, 100 KPa air pressure is required. In this case, as shown in Figure 14, compared to the actuator without the auxetic cavity structure, the maximum von Mise stress of the actuator with this structure was reduced by 189% during the working step. Therefore, the introduction of the auxetic cavity structure not only significantly improves the actuator’s deformation performance but also increases its service life.

## 4. Fabrication and Testing

### 4.1. Fabrication

**Figure 15:**
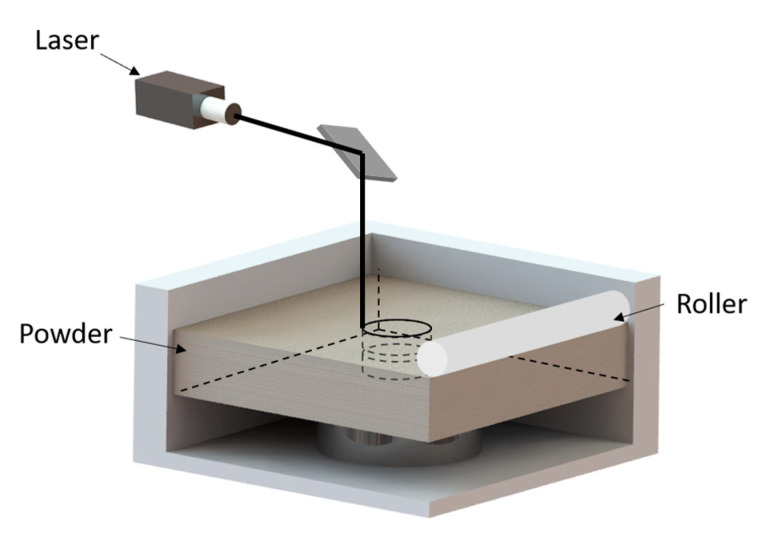
Schematic diagram of selective laser sintering manufacturing process.

In Figure 15, we present the manufacturing method for the soft actuators. We used selective laser sintering (SLS) of TPU powder for manufacturing.

Selective laser sintering is an additive manufacturing method in which an infrared laser is used as the energy source and a powder as the modeling material. During processing, the powder is first preheated to a temperature slightly below its melting point, and then the powder is laid flat under the action of the rollers; the laser beam is selectively applied for sintering under computer control based on the information of the layered cross-section, and after one layer is completed, the platform is lowered. The rollers again lay the next layer of powder flat and sinter it, and the excess powder is removed after the sintering is completed to obtain the final part.

The most significant advantage of the SLS process is the wide selection of materials, such as nylon, wax, ABS, metal, and ceramic powder, that can be used as sintering objects. In this design, we chose TPU powder. Since TPU powder has high elasticity and stiffness characteristics, the soft actuator can achieve large elastic deformation after sintering. At the same time, the high stiffness of the TPU actuator will increase the stability and load capacity of the robot working on vertical walls. 

Secondly, during the manufacturing process using the SLS method, the un-sintered part of the powder bed becomes a support structure for the sintered part, so there is no need to consider a support system. This is highly suitable for the actuator with the channel structure designed in this paper.

**Figure 16:**
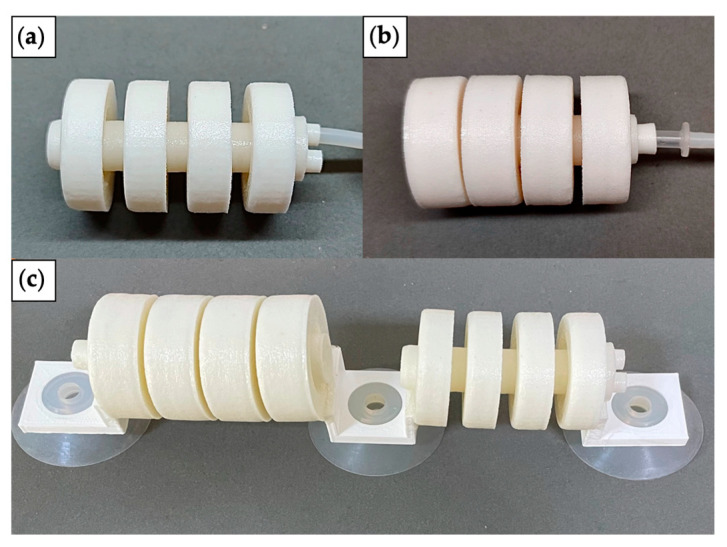
The fabricated models. (**a**) Single-channeled actuator. (**b**) Double-channeled actuator. (**c**) The robot prototype.

Figure 16a,b show the fabricated single-channeled and double-channeled actuators, respectively. The connecting devices are glued to the actuator, and the suckers are attached to the connecting devices by interference fit.

Sucker attachment is used as the anchoring method for the robot in this study. The suction force of the suckers translates into friction between the suckers and the contact surface, which is a critical factor in the robot’s ability to crawl steadily on a slope or vertical wall without slipping and is influenced by the coefficient of friction and the size of the suckers. Finding the right suckers and negative pressure air pump is a difficult process to analyze quantitatively, so using a testing method is more straightforward. 

The suckers we chose can carry a much larger load than the robot’s weight when attached vertically to glass, wooden materials, etc. The more considerable load margin does not affect the motion of the robot, so it was concluded that these suckers are suitable.

### 4.2. Testing of the Actuators

**Figure 17:**
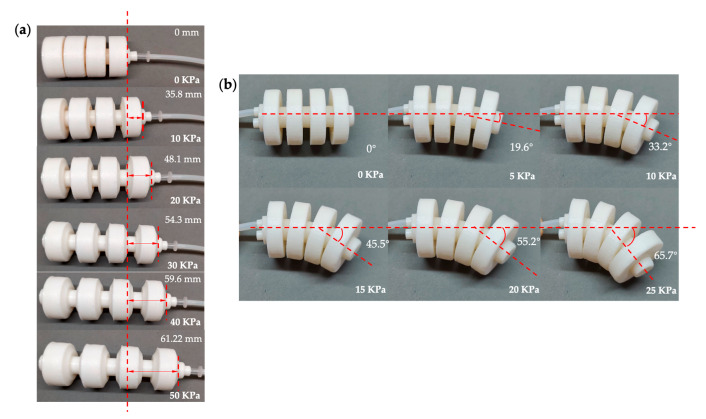
Testing of the actuators. (**a**) Single-channeled actuator. (**b**) Double-channeled actuator.

The actuators are connected to the air pumps and proportional valves through the air pipes. Figure 17a shows the elongation of the inflated single-channeled actuator under air pressure of 0–50 KPa. Figure 17b shows the bending angle of the inflated double-channeled actuator under air pressure of 0–25 KPa.

**Figure 18:**
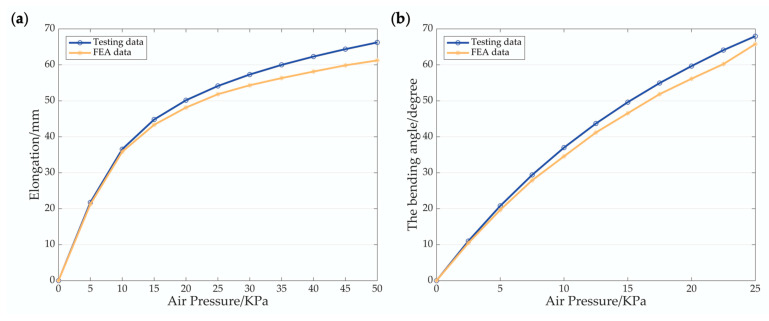
Comparison of the FEA data and the testing data. (**a**) Elongation of the single-channeled actuator. (**b**) Bending angle of the double-channeled actuator.

Finite element analysis data and test data are shown in Figure 18, including the elongation of the single-channeled actuator and the bending angle of the double-channeled actuator. The FEA data and the testing data are similar in tendency, and the maximum difference between the former and the latter is within 10% in numerical value. When the air pressure is low, the difference between the two is not significant, and as the air pressure gradually rises, the difference between the two increases significantly. There are two reasons for this result. Firstly, the Mooney–Rivlin three-parameter model of hyperelasticity chosen cannot wholly and accurately characterize the TPU material used. Secondly, there may be errors in the manufacturing process, resulting in differences between the actual actuator and the 3D model.

Considering the finite element analysis and testing data, 30 KPa for the single-channeled actuator and 20 KPa for the double-channeled actuator were used as the service pressure values.

### 4.3. Testing of the Robot Prototype

**Figure 19:**
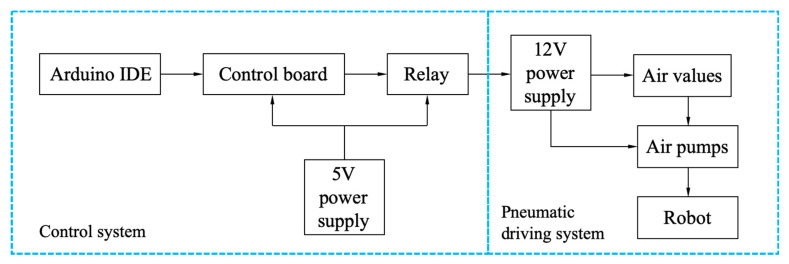
The control scheme diagram for the testing of the robot prototype.

The control scheme of the soft crawling robot, as shown in Figure 19, includes the control system and the pneumatic driving system. The Arduino IDE at the computer side sends the program to the Arduino Uno control board through the data line, and the control board transmits the signal to the relay, which controls the operation of the air pump and air solenoid valve to achieve robot movement. In addition, the control board and relays are powered by a 5 V battery, and a 12 V battery powers the air pump and air solenoid valve.

**Figure 20:**
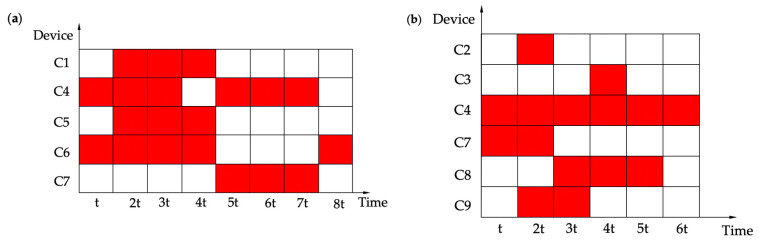
Hardware device working or idle diagram. (**a**) Straight-line locomotion. (**b**) Turning locomotion.

To maintain the coherence of the robot’s motion, it is necessary to ensure that the individual hardware devices cooperate. In Table 1, C1–C9 represent different devices, respectively. As shown in Figure 20, the hardware devices are demonstrated as working or idle during one cycle. The red indicates that the hardware device is working, and the white indicates that the hardware device is idle. *t* is the variable unit time, which can be modified according to the testing situation.

According to the control scheme, the circuit control system and the pneumatic drive system were built. Considering the response speed of the air pumps, the unit variable time was set to 1 s.

**Figure 21:**
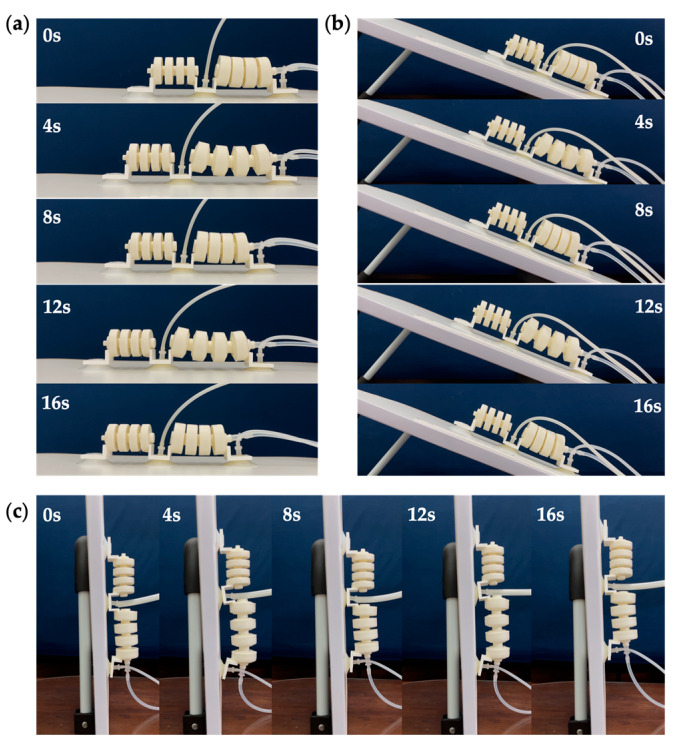
Straight-line locomotion testing. (**a**) On the flat ground. (**b**) On the slope. (**c**) On the vertical wall.

Figure 21 shows the straight-line locomotion testing of the robot prototype, which can crawl on flat ground, slopes, and vertical walls. A more powerful vacuum pump was used during vertical wall crawling to ensure suction power. In one cycle, the step length of the robot is 57.2 mm, so the straight-line crawling speed of the robot is approximately 7.15 mm/s. If the inflation rate of the air pump can be raised, then the variable unit time will be shortened, and the crawling speed of the robot will be increased.

**Figure 22:**
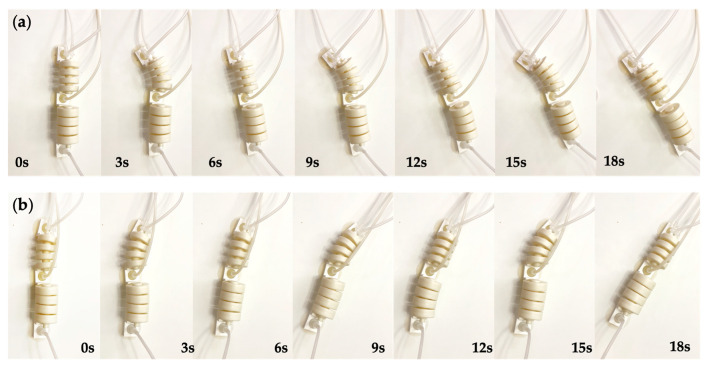
The turning locomotion testing. (**a**) Right turning. (**b**) Left turning.

Figure 22 shows the turning testing of the prototype robot, including left and right turning. The turning angle of the robot was 28.8 degrees in one cycle at the service air pressure. The significant difference between the turning angle and the actuator bending angle is that the friction between the suckers and the ground needs to be overcome. Thus, the inflation air pressure of the double-chamber actuator can be increased appropriately.

**Figure 23:**
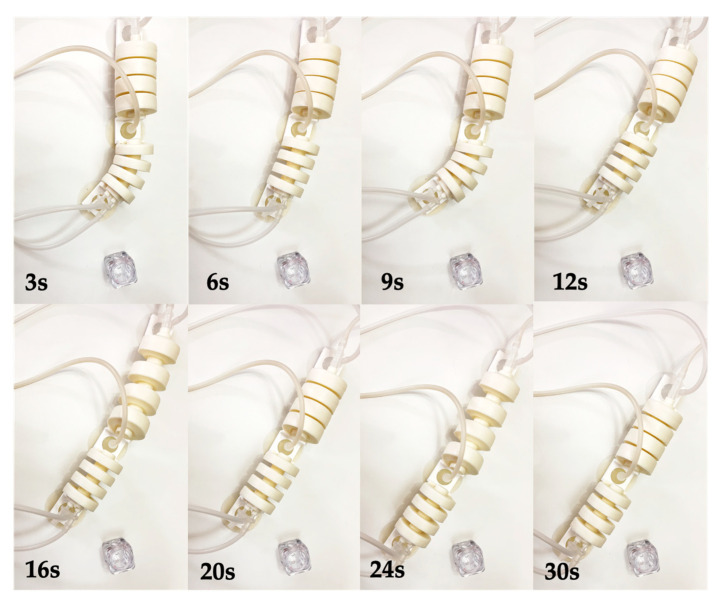
Obstacle avoidance testing of the robot.

By combining the turning and straight-line crawling processes, obstacle avoidance of the robot can be achieved, as shown in Figure 23.

**Figure 24:**
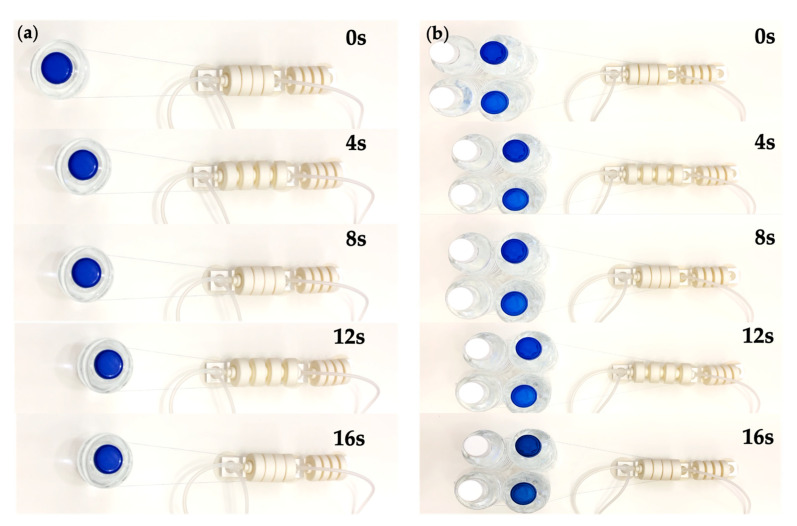
Load carrying capacity testing of the robot. (**a**) 500 g load. (**b**) 2000 g load.

**Figure 25:**
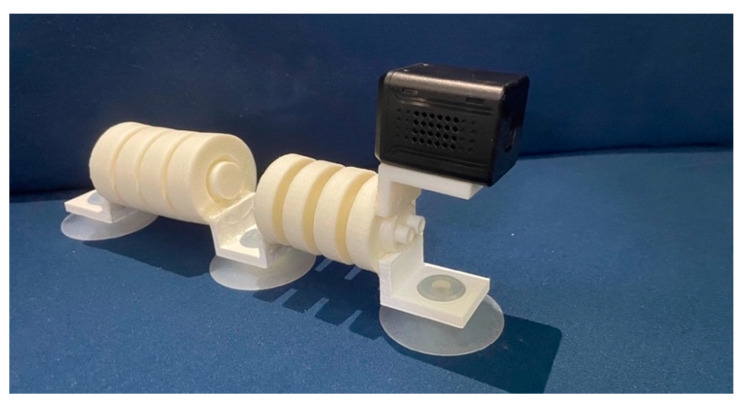
Camera installed on the robot.

Loads were attached to the robot, as shown in the Figure 24, and we let the robot drag them. On flat ground, the robot can pull heavy objects that are almost 20 times larger than its own mass. In future applications, we could utilize its equipment carrying capacity to a greater extent. Figure 25 shows the camera module being equipped on the robot in order to combine functions such as visual detection and recognition. A variety of testing of the robot is shown in Supplementary Video S1.

### 4.4. COT Discussion

Comparing the locomotor efficiency of various robots and organisms is possible using the dimensionless metric COT (cost of transport). It is computed by dividing the system’s average power input $P_{in}$ by the mass m, local gravitational acceleration g, and average speed v. It explains the energy cost of moving a given mass and distance:

(36) $$COT=\frac{P_{in}}{mgv}$$

The average power drawn from the pumps and valves in one straight motion cycle:

(37) $$P_{in}=\frac{3tP_{C3}+6tP_{C4}+3tP_{C5}+5tP_{C6}+3tP_{C7}}{8t}$$

where C3−C7 are the symbols shown in Table 1. 

Known that, $P_{C3}=P_{C4}=1\;W$, $P_{C5}=P_{C6}=P_{C7}=0.5\;W$, v=7.15 mm/s,

(38) $$m=m_{\max}=m_{robot}+m_{load}=2117.9\;g$$

Then,

(39) $$COT_{\min}=\frac{P_{in}}{mgv}=12.20$$

The robot in this paper has a lower COT value than some other bionic worm crawling robots, particularly rigid-body robots. This means that the robot in this paper can perform the same motion with less energy.

## 5. Conclusions and Discussion

In this study, a worm-like soft robot based on selective laser sintering of TPU powder was designed, analyzed, and fabricated. Through testing, we verified the feasibility of the prototype for flat ground locomotion, slope locomotion, vertical wall motion, and turning locomotion.

In terms of structure, inspired by worms, single-channeled and double-channeled pneumatic actuators with the auxetic cavity structure were proposed and designed in this paper, in order to achieve elongation and bending, respectively. The FEA method demonstrated that the von Mises stress at the stress concentration is reduced by 189% compared to the actuators without an auxetic cavity structure at the working step. According to the material failure theory, the structural design reduced the probability of crack appearance and growth and improved the service life of the actuators.

In terms of fabrication, this paper utilized the redundant powder’s own internal support based on selective laser sintering technology to fabricate the cavity structure of the soft actuator and improve its fabrication accuracy. At the same time, TPU powder was innovatively used to sinter and manufacture pneumatic actuators with significant stiffness. Compared with the commonly used silicone elastomer actuators, the TPU powder-sintered actuators are less affected by gravity. They can better achieve stability during crawling in different environments, especially on walls.

In terms of function, this paper accomplished the design goal of locomotion on flat ground, slopes, and vertical walls well through the coordination of soft actuators and vacuum suckers. After testing the prototype, the working air pressure of this robot was found to be 30 KPa for single linear motion, the standard step length was 57.2 mm under a working air pressure, and the average speed was 7.15 mm/s. The working air pressure for a single turn was 20 KPa, and the turning angle was 28.8 degrees under the working air pressure. Furthermore, the robot has a strong loading capacity for heavy objects. In the testing, we found that the robot can drag objects of 2000 g on a flat surface. 

For this robot, we propose three application scenarios:

1. Crawling on the inner walls of large containers, such as tanks;
2. Crawling on the wings and tails of passenger aircraft;
3. Crawling on glass curtain walls in high-rise buildings in order to clean them.

For application case 1, the tanks are prone to material failure via corrosion due to long-term exposure to liquid and gas mixing conditions. Since the surface curvature of large containers is great, this design can realize crawling in such scenarios and can carry detection equipment such as small cameras. However, due to the limitation of surface roughness, the use of an anchoring method based on suckers may not be stable. Therefore, when applied to non-smooth metal surfaces, the suckers can be considered to replace the electromagnetic anchoring method with variable stiffness. For application case 2, in order to ensure flight safety, airlines need to regularly test the wings and tails of passenger planes. Since these components have a large tilt angle and smooth surface, the robot’s design can be used to realize crawling on the wings and tails of the aircraft while carrying small nondestructive testing equipment. For application case 3, the designed robot can drag and carry a cleaning device containing a spray-cleaning agent with two parallel prototypes to clean key locations on glass curtain walls in high-rise buildings. In conclusion, the design can be adapted to more diverse environments and will have a wider range of industrial applications after the replacement of the anchor modules and by taking into consideration their passive compliant deformation.

## Figures and Tables

**Table 1:** The symbols for the different hardware devices.

Symbol	Hardware Device
C1	Air pump for single-channeled actuator
C2	Air pump for left channel of double-channeled actuator
C3	Air pump for right channel of double-channeled actuator
C4	Vacuum pump for the suckers
C5	Air solenoid valve for single-channeled actuator
C6	Air solenoid valve for the sucker at the back end
C7	Air solenoid valve for the sucker in the middle
C8	Air solenoid valve for the sucker at the front end
C9	Air solenoid valve for the double-channeled actuator

## Data Availability

Not applicable.

## Supplementary Materials

The following supporting information can be downloaded at: https://www.mdpi.com/article/10.3390/biomimetics7040205/s1, Video S1: Testing of the robot.
